# Longitudinal study of DNA methylation during the first 5 years of life

**DOI:** 10.1186/s12967-016-0913-x

**Published:** 2016-06-03

**Authors:** Rocio G. Urdinguio, María Isabel Torró, Gustavo F. Bayón, Julio Álvarez-Pitti, Agustín F. Fernández, Pau Redon, Mario F. Fraga, Empar Lurbe

**Affiliations:** Cancer Epigenetics Laboratory, Institute of Oncology of Asturias (IUOPA), HUCA, Universidad de Oviedo, Oviedo, Spain; Servicio de Pediatría, Consorcio Hospital General Universitario, Universidad de Valencia, Avda. Tres Cruces s/n, 46014 Valencia, Spain; CIBER Fisiopatología Obesidad y Nutrición (CB06/03), Instituto de Salud Carlos III, Madrid, Spain; Nanomaterials and Nanotechnology Research Center (CINN)-Spanish Council for Scientific Research (CSIC), (CINN-CSIC), Avenida de la Vega 4-6, 33940 El Entrego, Spain

## Abstract

**Background:**

Early life epigenetic programming influences adult health outcomes. Moreover, DNA methylation levels have been found to change more rapidly during the first years of life. Our aim was the identification and characterization of the CpG sites that are modified with time during the first years of life. We hypothesize that these DNA methylation changes would lead to the detection of genes that might be epigenetically modulated by environmental factors during early childhood and which, if disturbed, might contribute to susceptibility to diseases later in life.

**Methods:**

The study of the DNA methylation pattern of 485577 CpG sites was performed on 30 blood samples from 15 subjects, collected both at birth and at 5 years old, using Illumina^®^ Infinium 450 k array. To identify differentially methylated CpG (dmCpG) sites, the methylation status of each probe was examined using linear models and the Empirical Bayes Moderated *t* test implemented in the *limma* package of *R/Bioconductor*. Surogate variable analysis was used to account for batch effects.

**Results:**

DNA methylation levels significantly changed from birth to 5 years of age in 6641 CpG sites. Of these, 36.79 % were hypermethylated and were associated with genes related mainly to developmental ontology terms, while 63.21 % were hypomethylated probes and associated with genes related to immune function.

**Conclusions:**

Our results suggest that DNA methylation alterations with age during the first years of life might play a significant role in development and the regulation of leukocyte-specific functions. This supports the idea that blood leukocytes experience genome remodeling related to their interaction with environmental factors, underlining the importance of environmental exposures during the first years of life and suggesting that new strategies should be take into consideration for disease prevention.

## Background

DNA methylation is an epigenetic mechanism that regulates different genome functions, including gene expression, which may intervene in physiological events such as cell lineage determination, cell differentiation, cell maturation and tissue-specific gene expression [1, 2]. Much of a person’s epigenomic pattern is established during embryogenesis and early development of the fetus [3]. However, genomic DNA methylation is known to be sensitive to environmental stimuli and changes during lifetime and with aging [4]. Some epigenomic modifications over time are important in development, but others occur stochastically [5, 6]. These alterations in DNA methylation patterns have been suggested to account for many age-related diseases [7–10]. For instance, age-associated alterations in DNA methylation have been found to be involved in the initiation and progression of cancer and certain chronic diseases [11].

The relationship between DNA methylation levels and age has already been demonstrated [12–16]. In fact, the use of DNA methylation data has been proposed as a method of measuring biological aging and it is possible to predict the age of a tissue based on its methylation pattern at specific CpG sites [12, 13, 17–19]. However, most studies exploring age-associated DNA methylation changes have been carried out on adults and have focused on aspects such as cell senescence, longevity, cancer, stem cell functions and chronological age [18, 20–25]. Reports on DNA methylation patterns during early childhood are still scarce [26–30]. The characterization of DNA methylation patterns during the first years of life is an ongoing task, and data from longitudinal studies are more revealing. DNA methylation levels have been shown to change rapidly during early development, with more pronounced changes in the immediate postnatal years, while methylation levels at many sites tend to stabilize beyond age 7 [31].

Additionally, early life conditions can predispose the fetus to a range of adult health outcomes, and DNA methylation seems to play an important role in this process [32, 33]. For instance, the time immediately before and after birth may be a sensitive period related to programming cardiometabolic risk [34, 35]. Adult health outcomes are therefore determined not only by conventional risk factors experienced in adult life, but also by early life programming [36], which has been shown to be mediated by DNA methylation [37].

Due to the influence of early life epigenetic programming on health outcomes and the fact that DNA methylation levels seem to change more rapidly during the first years of life, the identification of CpG sites that are modified by age in infants would lead to the detection of genes that might be epigenetically modulated by environmental factors during early childhood. If disturbed, these might contribute to susceptibility to specific diseases later in life [38, 39].

Thus, the aims of this study were: (1) the identification of CpG sites with changes in DNA methylation levels measured longitudinally between cord blood samples and peripheral blood samples at 5 years after birth in a group of 15 children. Children who were small (SGA), appropriate (AGA), and large for gestational age (LGA), and normal weight or overweight/obese at 5 years old were included; and (2) the characterization of the genomic distribution and functional relationships of age-modified CpG sites during early childhood.

## Results

**Fig. 1:**
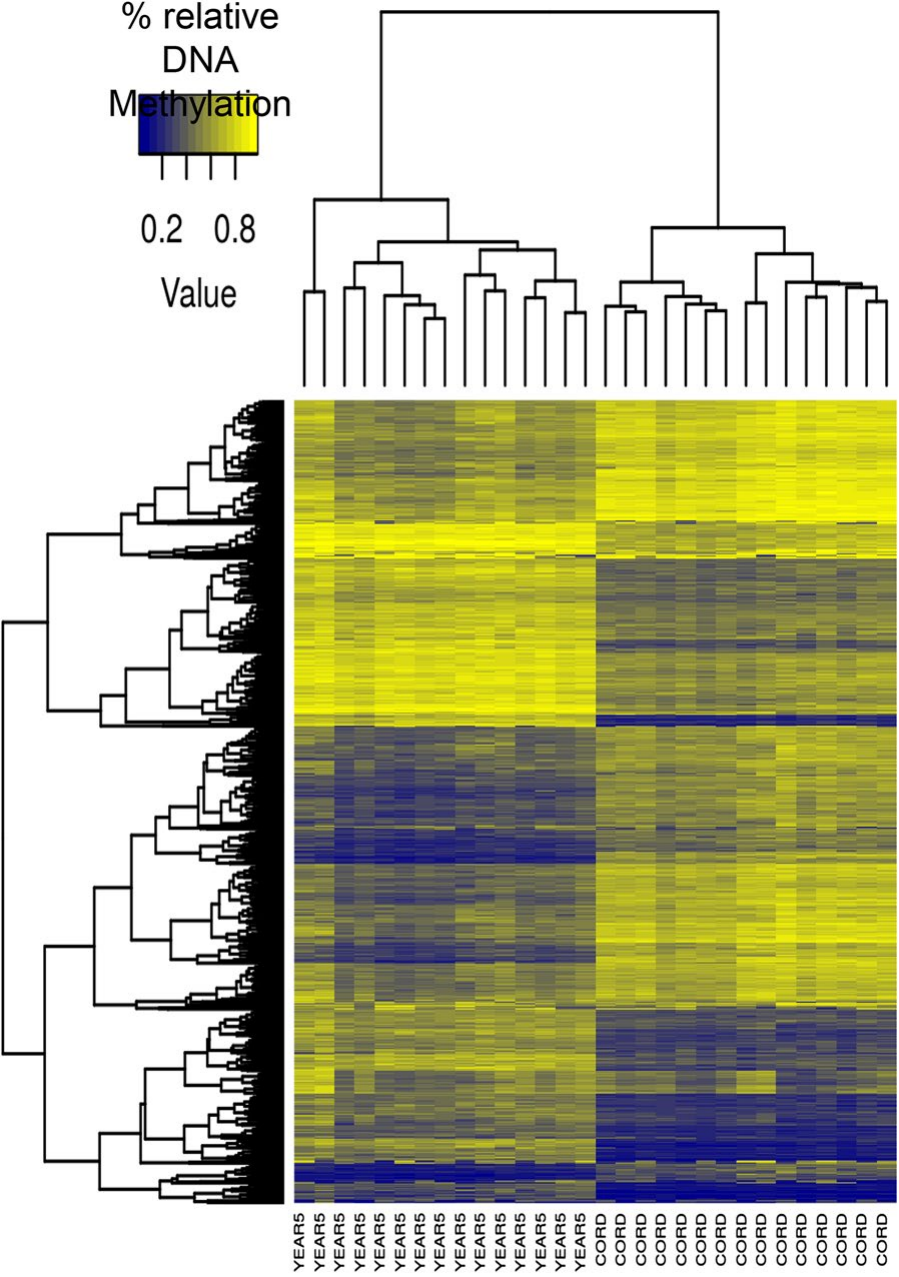
Clustered heatmap showing the methylation levels across all samples for the hyper- and hypo-methylated CpG sites. Methylation levels range from *dark blue* (no methylation) to *light yellow* (100 % methylated). Dendrograms were computed using Euclidean distance and a complete cluster agglomeration method. Both age groups are clustered correctly and methylation values have a homogeneous intra-group profile. YEAR5 stands for samples of DNA isolated from blood that was collected from individuals when they were 5 years-old. CORD stands for samples of DNA isolated from cord blood that was collected at birth from the same individuals

In order to identify DNA methylation changes with time during the first 5 years of life, the methylation patterns of 484103 CpG sites in cord and 5-year-old blood samples from the same 15 subjects were compared. We found that the DNA methylation levels of 6641 CpG sites changed as a function of age. Specifically, 2443 probes (36.79 %) were hypermethylated with time, corresponding to 1407 genes; and 4198 probes (63.21 %) corresponding to 2640 genes, were hypomethylated with time. Hierarchical clustering of all samples using the dmCpGs enabled each sample to be correctly classified into its corresponding age group (Fig. 1).

**Fig. 2:**
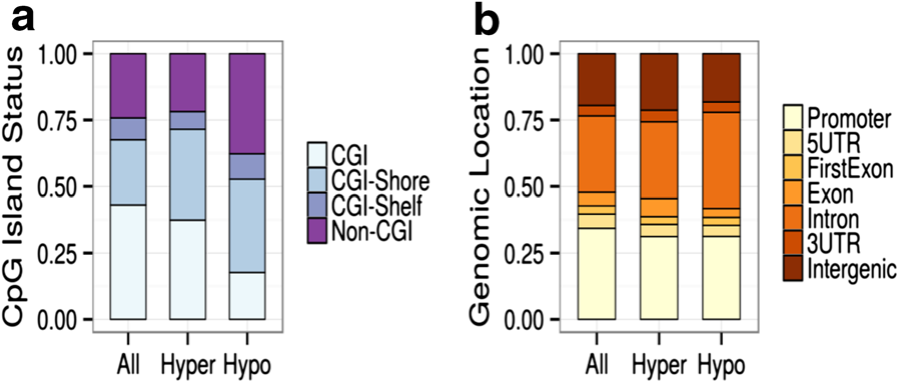
Genomic characterization of the dmCpGs with time. **a** Stacked *bar chart* describing the proportion of CpG sites in the selected subsets of interest according to their CpG Island status and relative to the background Illumina^®^ 450 k (*All*) proportions. Hypermethylated probes are enriched in CpG island shores while hypomethylated CpG sites are enriched in non CpG islands (CGIs) (Pearson’s Chi squared test; p < 0.001, OR = 1.60 and p < 0.001, OR = 1.91, respectively). **b** Stacked *bar chart* showing the proportion of selected CpG sites with respect to their genomic location and relative to the background (*All*). Hypermethylated CpG sites are enriched mainly in exons (Pearson’s Chi squared test; p < 0.001, OR = 1.37), and hypomethylated probes in introns (Pearson’s Chi squared test; p < 0.001, OR = 1.41)

**Fig. 3:**
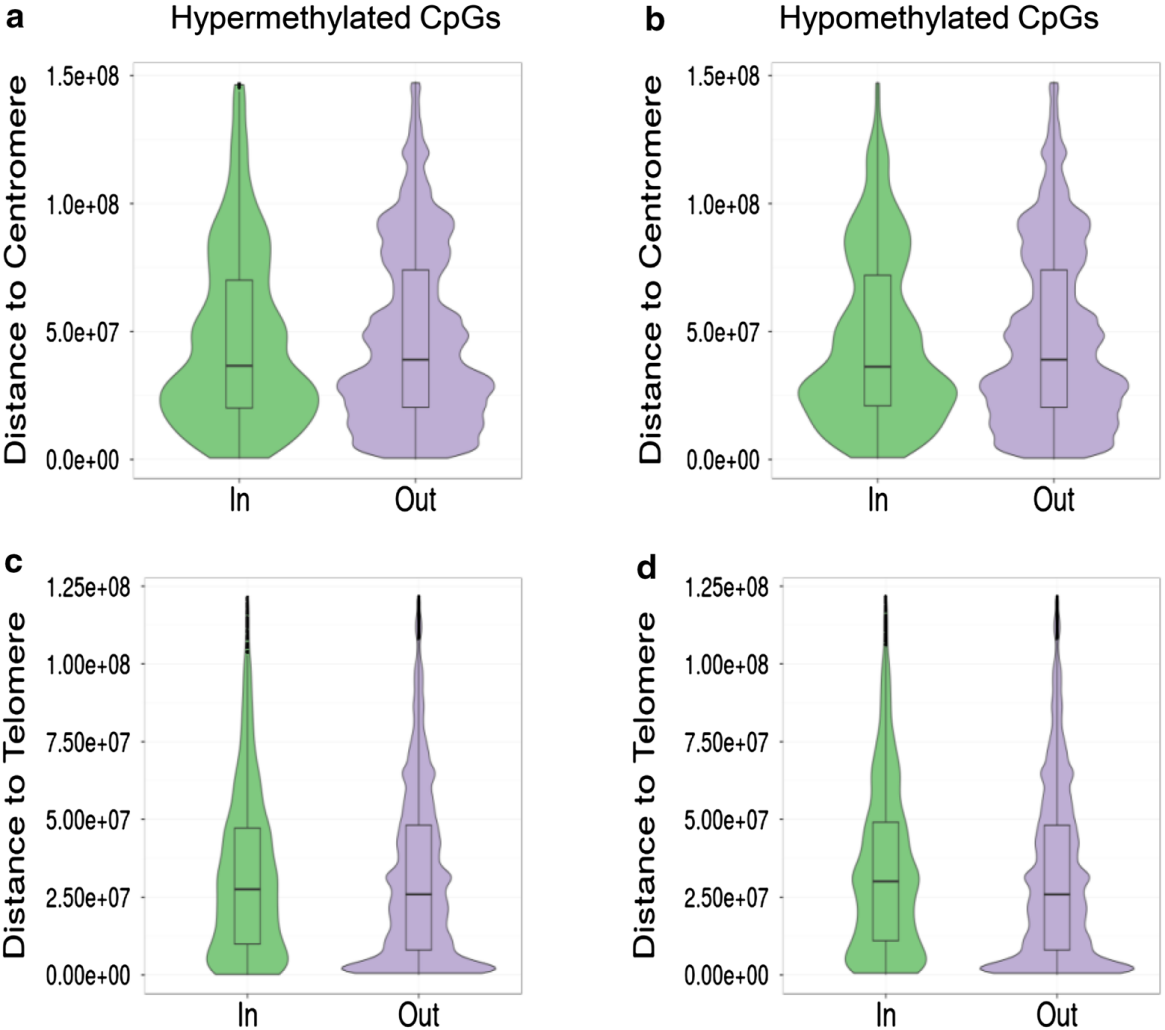
Distance to centromere (**a**, **b**) and telomere (**c**, **d**) of differentially methylated probes. **a**, **b** *Violin plots* showing the distribution of the distance to centromeres for the hypermethylated (**a**) and hypomethylated (**b**) CpG sites (*In*) with respect to those sites belonging to the Illumina^®^ 450 k microarray but not included in the corresponding subset of interest (*Out*). There is no statistically significant relationship between both hyper- and hypomethylated CpG sites and their distance to centromeres. **c**, **d** *Violin plots* showing the distribution of the distance to telomeres for the hypermethylated (**c**) and hypomethylated (**d**) CpG sites (*In*) with respect to those sites belonging to the Illumina^®^ 450 k microarray but not included in the corresponding subset of interest (*Out*). Only hypomethylated probes have a statistically significant change in their distance to telomeres, but with a minimal effect size (Wilcoxon test; p < 0.001, D = −0.0017)

To characterize these dmCpG sites from a functional genomics point of view, we first determined their distribution within the different regions of the CpG islands [40]. Hypermethylated probes were enriched in CpG island shores, while hypomethylated CpG sites were enriched in non CpG islands (CGIs) (Pearson’s Chi squared test; p < 0.001, OR = 1.60 and p < 0.001, OR = 1.91, respectively) (Fig. 2a). In terms of genomic location, hypermethylated CpG sites were enriched mainly in exons (Pearson’s Chi squared test; p < 0.001, OR = 1.37), and hypomethylated probes in introns (Pearson’s Chi squared test; p < 0.001, OR = 1.41) (Fig. 2b). There was no statistically significant relationship between both hyper- and hypomethylated CpG sites and their respective distances to centromeres. On the other hand, only hypomethylated probes have a statistically significant change in their distance to telomeres (Fig. 3), but with a minimal effect size measured by Cliff’s Delta (D) (Wilcoxon test; p < 0.001, D = −0.0017), which seems to be non-biologically relevant.

**Fig. 4:**
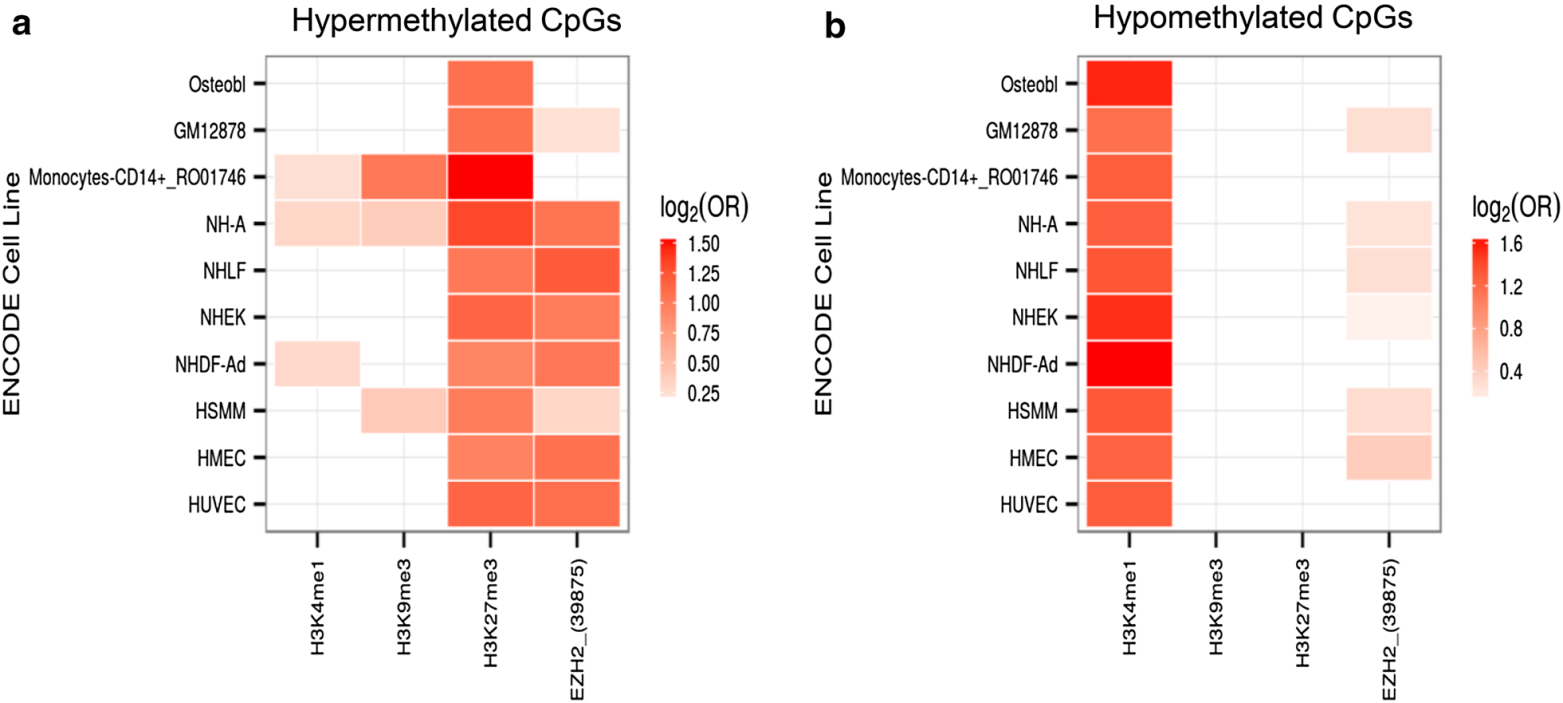
*Heatmaps* showing the association between the location of hypermethylated (**a**) and hypomethylated (**b**) CpG sites and enriched regions for several chromatin marks and cell lines. Chromatin marks peak location information for each cell line was extracted from the ENCODE BROAD Histone project information available at the UCSC Genome Browser. Associations between CpG site and chromatin mark peak locations were tested using a Fisher's exact test. P values were adjusted for multiple comparisons and only those falling under a 0.05 FDR threshold are shown as *colored spots* in the heatmap. The base-2 logarithm of the odds ratio (OR) was used as a measure of effect size. Associations with higher effect sizes are drawn in *darker shades of red*. There are statistically significant associations of hypermethylated (**a**) CpGs with the repressive histone mark H3K27me3 and the polycomb group protein EZH2 in most differentiated ENCODE cell lines. On the other hand, hypomethylated probes are associated with regions enriched in H3K4me1 (**b**)

To distinguish the chromatin marks associated with the dmCpG sites showing changes over time, the DNA sequences identified in our study were analyzed against previously reported data on a collection of histone modifications and chromatin modifiers in 10 different cell types obtained from healthy individuals, (see “Methods” section), where hematologic cells are also represented. In the present study, we found statistically significant associations of hypermethylated CpGs with the repressive histone mark H3K27me3 and the polycomb group protein EZH2 in most differentiated ENCODE cell lines (Fisher's exact test; p < 0.05) (Fig. 4). This is in line with previously published data [41, 42]. Similarly, hypomethylated probes were here associated with regions enriched in H3K4me1 (Fisher's exact test; p < 0.05) (Fig. 4). This has been shown previously, where age-associated changes of DNA methylation were studied in differentiated and adult stem cells [41].

The analysis of the gene ontology (GO) of the genes associated with the differentially methylated probes showed that both hyper- and hypomethylated genes were significantly enriched (FDR <0.05) in specific GO terms of biological processes, molecular functions and cellular components (Tables 1, 2). Hypermethylated genes were associated with biological processes related to development and cell adhesion, with molecular functions related to sequence-specific DNA binding, and cellular components such as dendrite or axon (Table 1). On the other hand, hypomethylated genes were associated with biological processes related to immune system regulation, with molecular functions related to antigen binding and intracellular signalling, and with cellular components related to the MHC protein complex and cytoskeleton (Table 2).

**Table 1:** Gene ontology analysis of hypermethylated GpG sites from birth to 5 years of age (with RR >2 and Q Value <0.001)

Onthology	Term	RR	Q_value
Biological_process	Digestive tract morphogenesis	6.3551	2.25E−05
Biological_process	Homophilic cell adhesion	4.6179	7.02E−09
Biological_process	Digestive tract development	3.6972	0.0001
Biological_process	Locomotory behavior	3.6791	1.77E−07
Biological_process	Cell–cell adhesion	3.6197	1.63E−07
Biological_process	Digestive system development	3.3312	0.00035
Biological_process	Hindbrain development	3.1444	0.00072
Biological_process	Cell fate commitment	2.7058	0.00011
Biological_process	Behavior	2.4935	2.83E−08
Biological_process	Embryonic organ morphogenesis	2.4633	0.00027
Biological_process	Single-organism behavior	2.3998	3.88E−05
Biological_process	Cell adhesion	2.3899	4.55E−13
Biological_process	Regionalization	2.381	0.00015
Biological_process	Biological adhesion	2.3795	4.55E−13
Biological_process	Brain development	2.2605	4.09E−08
Biological_process	Pattern specification process	2.2365	5.37E−05
Biological_process	Central nervous system development	2.2029	5.18E−10
Biological_process	Muscle structure development	2.1471	0.00017
Biological_process	Embryonic morphogenesis	2.1396	1.59E−05
Biological_process	Embryonic organ development	2.0979	0.00047
Biological_process	Skeletal system development	2.0909	0.00035
Molecular_function	Sequence-specific DNA binding	2.0117	2.71E−06
Cellular_component	Dendrite	2.3717	1.38E−05
Cellular_component	Axon	2.2741	0.00099
Cellular_component	Somatodendritic compartment	2.1637	4.07E−06

*RR* relative risk is a measure of effect size describing the change of proportions between our selected set of genes and a given term

*Q value* Q value is the result from the adjustment of P values in order to control the false discovery rate (FDR) using the Benjamini-Hochberg method

**Table 2:** Gene ontology analysis of hypomethylated GpG sites from birth to 5 years of age (with RR >2 and Q value <0.01)

Onthology	Term	RR	Q_value
Biological_process	Stress fiber assembly	23.288	0.00494
Biological_process	Positive regulation of T cell mediated immunity	4.9028	0.0066
Biological_process	Regulation of T cell mediated immunity	4.7907	0.00156
Biological_process	Positive regulation of cell killing	4.4359	0.00494
Biological_process	Response to type I interferon	3.502	0.00586
Biological_process	Interferon-gamma-mediated signaling pathway	3.3269	0.00653
Biological_process	Maintenance of protein location in cell	3.0629	0.00181
Biological_process	Cellular response to interferon-gamma	2.9322	0.00494
Biological_process	Maintenance of location in cell	2.9052	0.00191
Biological_process	Response to interferon-gamma	2.6615	0.00496
Biological_process	Actin cytoskeleton organization	2.0415	9.66E−05
Biological_process	Actin filament-based process	2.0231	3.32E−05
Molecular_function	Antigen binding	3.8842	0.00174
Molecular_function	Rho guanyl-nucleotide exchange factor activity	3.3577	0.00237
Molecular_function	Ras guanyl-nucleotide exchange factor activity	3.0522	0.00023
Molecular_function	Guanyl-nucleotide exchange factor activity	2.2431	0.00345
Cellular_component	MHC class I protein complex	11.685	0.00637
Cellular_component	MHC protein complex	7.2337	0.0005
Cellular_component	Integral component of lumenal side of endoplasmic reticulum membrane	4.8966	0.00844
Cellular_component	Lumenal side of membrane	4.8966	0.00844
Cellular_component	Lumenal side of endoplasmic reticulum membrane	4.8966	0.00844
Cellular_component	Main axon	3.9646	0.00124
Cellular_component	Cortical cytoskeleton	3.0207	0.00807
Cellular_component	Axon part	2.4846	0.00206
Cellular_component	Ruffle	2.1639	0.00663

*RR* relative risk is a measure of effect size describing the change of proportions between our selected set of genes and a given term

*Q value* Q value is the result from the adjustment of P values in order to control the false discovery rate (FDR) using the Benjamini-Hochberg method

To analyze whether methylation changes with time were associated with birth weight or being overweight at 5 years of age, the comparative analysis was performed considering these two variables. No significant DNA methylation changes were found in relation to SGA group, or to being overweight at 5 years old (normal weight/overweight). It was not possible to determine whether there was an association between the SGA and being overweight at 5 years old, due to the fact that none of the individuals that were overweight at 5 years old belonged to the group of subjects who were SGA.

## Discussion

The present longitudinal study focuses on the dynamics and the context of DNA methylation changes during early childhood in peripheral blood leukocytes. Data were compiled from 30 blood samples corresponding to 15 individuals at two time points (umbilical cord at birth, and 5 years after birth). It was shown that DNA methylation levels are modified as a function of age in 6641 CpG sites, most of them being hypomethylated. In hyper- and hypomethylated CpG sites, DNA methylation changes were significantly associated with intragenic regions, with exons and introns respectively. This implies that these DNA methylation changes are non-randomly distributed and specifically occur in discrete regions of the genome.

To further examine the features of the identified dmCpGs, the GO terms related to the genes associated with the differentially methylated probes were characterized. Both hyper- and hypomethylated sites were significantly enriched in specific GO terms of biological processes, molecular functions and cellular components. Specifically, it was found that genes with age-hypermethylated CpG sites were enriched in biological processes related to different tissue morphogenesis and development. This is in line with previous studies where increased DNA methylation was involved in silencing developmental genes [43].

Regarding hypomethylation with age, this study supports findings from previous reports where CpG sites, which are age-hypomethylated in the first 2 or 5 years following birth, are enriched in immune-related genes [26, 29]. Taking into account that a decrease in DNA methylation levels at promoter regions is known to enable gene expression [44], DNA methylation changes with age during the first years of life in human leukocytes may be closely associated with cell differentiation, and commitment to lymphoid and myeloid lineages [45]. These results thus denote that differences in DNA methylation associated with age may not only be triggered by stochastic DNA methylation changes [24, 46], but may also be related to the immune system function [26]. Additionally, age-hypomethylated CpGs sites were enriched in genes related to the MHC protein complex. This finding is in line with a recent study in samples from birth to 5 years old, where DNA methylation levels in class I and class II MHC molecules were found to decrease with age [26].

To explain the mechanisms that mediate the DNA methylation changes observed during aging, an increasing number of studies have focused on the identification of the factors determining the dynamics of DNA methylation. For instance, genes that are hypermethylated in blood during aging have been recently associated with the presence of bivalent chromatin domains in embryonic stem cells [21, 42, 47, 48], as well as with repressive histone marks (H3K27me3/H3K9me3) in differentiated cells [41, 42]. The results of the present study indicate the presence of the same repressive histone marks found in differentiated cells in the sequences that are hypermethylated with time during the first 5 years of life in leukocytes. This finding supports the notion that these repressive histone marks are related to DNA methylation gain during aging, independent of the type of cell or its potential, as previously described [41].

The present data also show a strong enrichment in the active chromatin mark H3K4me1 in age-hypomethylated sequences, which is in line with the data provided for hypomethylated sequences in MSCs and differentiated cells during aging [41]. This finding points towards this histone modification being of use as a cell-type-independent chromatin signature of DNA hypomethylation during aging. Additionally, a recent study indicated correspondence of H3K4me1 with enhancers [49], and an association between DNA hypomethylation within specific transposable elements and tissue-specific enhancer marks [50]. This suggests that H3K4me1-associated DNA hypomethylation could play a role in tissue-specific epigenetic gene regulation and the deregulation of gene expression during aging [51]. More studies, however, are required to clarify the mechanisms managing the methylation machinery to the age-modified loci during this time window.

A major issue in age-related DNA methylation studies is hematologic cell heterogeneity [52, 53], due to the fact that DNA methylation is usually measured in unfractionated blood. In order to adjust the model of analysis, a Surogate Variable Analysis (SVA)-based approach was applied, as described in Leek et al. [54] (see “Methods” section for details). This ensured that cell heterogeneity had a minimal impact on the blood DNA methylation data.

One limitation of the present study was that between-group differences with respect to variables such as SGA or being overweight at 5 years of age could not be adequately evaluated. This was due to the number of individuals belonging to each category analyzed being insufficient or too unequally distributed between groups to allow for comparisons and analysis. A larger number of individuals should be incorporated to a future study to address this issue.

**Fig. 5:**
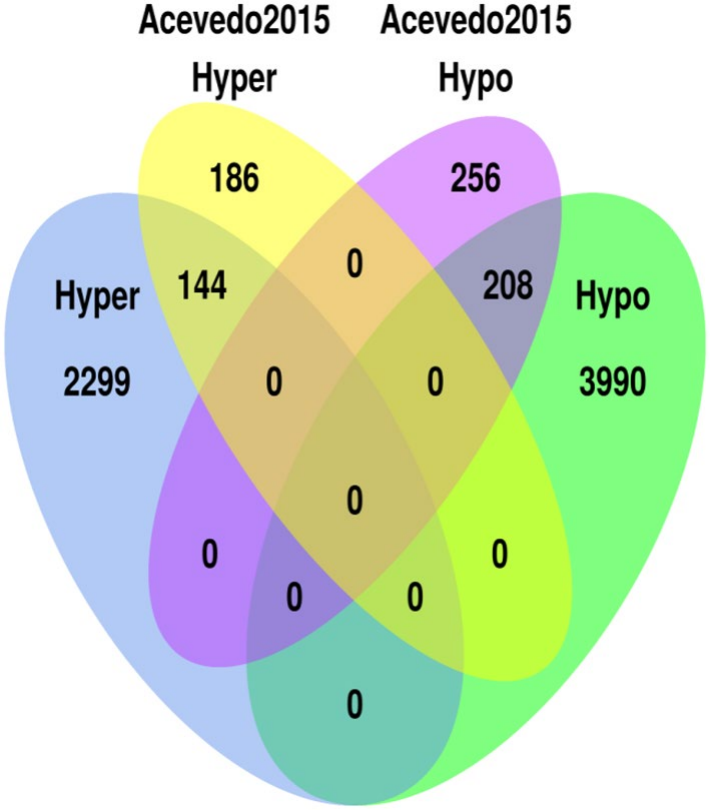
*Venn diagram* showing the intersections of the identified hyper- and hypo-methylated CpG sites and those described in Acevedo et al. [26]. There is a general consensus over the methylation direction of change between our results and the selected literature. Similarities and differences among the corresponding subsets are also shown

Several studies have explored DNA methylation patterns during early childhood [26–31]. For instance, blood samples from 3 months and 5 years of age were analyzed using the HumanMethylation450 BeadChip in the longitudinal study performed by Acevedo et al. [26]. This provided a total of 794 CpG sites where 330 CpG sites (41.5 %) were age-methylated and 464 CpG sites (58.4 %) were age-demethylated. When comparing with their results, it was found that 144 (43.64 %) of their hypermethylated and 208 (44.83 %) of their hypomethylated probes were identified by the present study (Fig. 5). Common GO terms (related to MHC protein complex) were also found when analyzing probes that were hypomethylated in both studies (Table 2). Furthermore, the tendency of a loss of methylation with age was corroborated in our set of samples. Additionally, other studies have identified different regions with changes in DNA methylation with age [27–31]. The possible differences in the CpG sites found in the literature could be explained due to disparities in the cell type (buccal epithelium, mononuclear cells, blood…), methodologies (HumanMethylation450 and/or 27 BeadChip), ages included in the study, methods of analysis, or purpose of the studies, for instance.

## Methods

### Selection of participants

Parents of newborns born at term (gestational age ≥37 weeks) in the General Hospital, University of Valencia, Spain, after uncomplicated pregnancies and in the absence of perinatal illness were randomly invited to participate in the study. Exclusion criteria were multiple gestations, cesarean section, and that parents were planning to move out of the area after delivery. Gestational age at birth was ascertained according to the method of Ballard et al. [55], and the general characteristics of gestation and delivery for each participant were obtained from routine obstetrical records. Subjects were divided according to birth weight (BW) and gestational age—SGA, <10th percentile for their sex; AGA, between 10th–90th percentile; and LGA, >90th percentile [56]. The subjects were followed-up at 5 years of age and all measurements were taken at birth and at 5 years. At birth all parents gave informed consent for their children to participate in the study, and the Committee for the Protection of Human Subjects of the Hospital General approved the study according to the Declaration of Helsinki.

### Anthropometric parameters

At 5 years, body weight was recorded to the nearest 0.1 kg using a standard beam balance scale with the subjects wearing light indoor clothing and no shoes. Height was recorded to the nearest 0.5 cm using a standardized wall-mounted height board. Body mass index (BMI) and the corresponding standard deviation were calculated, with BMI being the weight in kilograms divided by the square of the height in meters. Subjects with a BMI ranging from the 85th to 95th percentile were defined as being overweight [57] while they were defined as obese when having a BMI above the 95th percentile [58].

In total, 15 subjects were enrolled in this study. Eight of the 15 subjects (53.3 %) (2 boys/6 girls) were SGA and the other seven (46.7 %) (4 boys/3 girls) were AGA or LGA. Four individuals were overweight/obese at 5 years old (BMI between 17 and 21.4), none of whom were SGA (Table 3).

**Table 3:** Clinical information of the 15 subjects enrolled in this study

		Information at birth						Information at year 5							
Sample	Sex	Sample group	Gestational age (weeks)	Lactation group	Birth weight group	Birth weight (g)	Birth length (cm)	Sample group	Age	Year 5 weight (g)	Year 5 height (cm)	Year 5 body Mass Index	Year 5 Z-score	Year 5 percentile	Year 5 overweight
1	Male	CORD	38	FF	SGA	2155	46.0	YEAR5	5	17,000	111.0	13.8	−1.70	4	No
2	Female	CORD	39	BF	SGA	2340	45.5	YEAR5	5	18,600	113.0	14.6	−0.50	31	No
3	Female	CORD	38	FF	SGA	2400	48.5	YEAR5	5	16,600	107.5	14.4	−0.69	25	No
4	Female	CORD	39	BF	SGA	2450	48.0	YEAR5	5	20,200	110.5	16.5	0.90	82	No
5	Female	CORD	37	BF	SGA	2540	49.0	YEAR5	5	22,500	116.0	16.7	1.00	84	No
7	Male	CORD	37	FF	SGA	2720	47.0	YEAR5	5	15,700	102.5	14.9	−0.65	26	No
8	Female	CORD	38	FF	SGA	2740	47.5	YEAR5	5	19,300	112.0	15.4	0.18	57	No
9	Female	CORD	39	FF	SGA	2770	46.5	YEAR5	5	15,000	103.0	14.1	−0.92	18	No
10	Female	CORD	38	FF	AGA	3540	49.5	YEAR5	5	26,100	118.5	18.6	1.57	94	Yes
11	Male	CORD	40	FF	AGA	3580	51.0	YEAR5	5	18,700	113.0	14.6	−0.64	26	No
13	Male	CORD	40	FF	AGA	3800	50.0	YEAR5	5	22,700	103.0	21.4	2.90	99	Yes
14	Female	CORD	39	BF	LGA	3840	49.5	YEAR5	5	24,000	112.0	19.1	1.92	97	Yes
15	Male	CORD	39	FF	LGA	3860	50.0	YEAR5	5	22,500	115.0	17.0	1.14	87	Yes
16	Female	CORD	39	BF	LGA	3940	51.0	YEAR5	6	20,000	115.0	15.1	−0.21	42	No
17	Male	CORD	40	BF	LGA	4380	51.0	YEAR5	5	22,000	114.3	16.8	0.95	83	No

*FF* formula feeding, *BF* breastfeeding, *SGA* small for gestational age, *AGA* appropriate for gestational age, *LGA* large for gestational age

### Sample collection, DNA extraction, and quantification

Blood samples were collected from 15 subjects at the two testing times. First, cord blood samples were taken at birth, and second, peripheral venous blood samples were taken from each child during their fifth year of life. Genomic DNA was extracted with the RealPure kit (RealPure, REAL, Durviz, Ref: RBMEG01) and quantified with the Nanodrop-2000C Spectrophotometer. A DNA quality check was performed with Quant-iT PicoGreen dsDNA reagent.

### Illumina^®^ Infinium 450 k data preprocessing

The study of the DNA methylation pattern of 485577 CpG sites was performed using Illumina^®^ Infinium 450 k array and the IDAT files from the microarray were processed further using the R/Bioconductor package *minfi* [59]. In order to adjust for the different probe design types present in the 450 k architecture, red and green signals from the IDAT files were corrected using the SWAN algorithm [60]. No background correction or control probe normalization was applied. Probes where at least two samples had detection p values over 0.01 were filtered out. In accordance with Du et al. [61], both Beta values and M values were computed and employed across the analysis pipeline. M values were used for all the statistical analyses, assuming homoscedasticity, while Beta values were mostly used for the intuitive interpretation and visualization of the results.

### Batch effect correction

Surrogate Variable Analysis (SVA) [54] was employed to capture the heterogeneity of the underlying methylation data and to account for possible batch effects or confounding variables that might be of interest. Coefficients for the detected surrogate variables (SVs) were later added to the phenotypical data and included in the definition of a model in order to detect differentially methylated probes (DMPs). The R/Bioconductor package *sva* [62] implementation was used to estimate the number of SVs and their coefficients, using both age group (newborns/five year olds) and gender as covariates of interest, and only one intercept term as a null background model. Multidimensional scaling (MDS) was employed as a visualization tool whenever there was a need to illustrate the influence of possible confounders on the data.

### White blood cell heterogeneity adjustment

Cellular heterogeneity is a main source of variation in Epigenomic studies [63]. Each cell type has a different Epigenomic profile, and variations of the different subpopulations can often be confounded with the phenotype of interest, resulting in a higher rate of both false positives and negatives. This is especially true when using whole blood as our main tissue, due to its highly variable subpopulation composition. This is especially relevant if we take into account the number of Epigenomic studies that have been published and that use whole blood as their main tissue.

One of the most common approaches to dealing with blood cellular heterogeneity is the Houseman method [53], which uses a methylation database of several, pure-lineage samples in order to compute an approximation to the real subpopulation percentages. Using this information, the method is able to adjust the original methylation dataset and generate a new one where the confounder influence has been removed. Several other methods have been proposed that expand on this concept, and some of them do not even require having a purified samples methylation database in advance [64].

However, there are also other approaches to the detection of confounding factors that do not need information about the Epigenomic profiles of the different cell subtypes. SVA [54], for example, is a general framework for the detection of structured variability patterns over the residuals of a previously fitted model using the main phenotype of interest. In general, SVA is not only able to capture the variation due to cellular heterogeneity, but also due to other factors, some of them possibly unknown to the researcher.

After an exploratory analysis of the data, it was decided to use SVA to capture the main confounding factors in our dataset, and to include them in our model. This resulted in a better fitted model than those based on the Houseman method alone, which suggested that in our case SVA is able to capture the cellular subpopulations proportion variations occurring in our data.

### Detection of differentially methylated probes

Significant methylation of a probe was determined by the moderated t test implemented in the R/Bioconductor package limma [65]. A linear model, with methylation level as response and all the combinations of age group, birth weight and overweight at 5 years as the main covariate of interest, was fitted to the methylation data. Surrogate Variables generated using SVA and information regarding the gender and pair ID of the samples was also included in the model definition. Contrasts were then defined as the linear combinations of the different values the main covariate of interest could take, in order to represent the different questions arising from the model design. Each contrast generated a coefficient and P value for each probe. P values were corrected for multiple testing using the Benjamini-Hochberg method for controlling the false discovery rate (FDR). A FDR threshold of 0.05 was employed to determine DMPs.

### Histone enrichment analysis

In order to analyze the enrichment of histone marks for a subset of probes, the information contained in the UCSC Genome Browser Broad Histone track from the ENCODE Project was used. Histone mark peaks were downloaded for every combination of cell line and antibody. For each track, a 2 × 2 contingency table was built to represent the partition of the whole set of possible probes in the microarray with respect to their membership of the subset of interest and the overlap between the probes and the histone peaks. A Fisher's exact test was used to determine whether there was a significant enrichment of the selected histone mark for the subset of interest. P values were adjusted for multiple comparisons using the Benjamini-Hochberg method for controlling the FDR. A significance level of 0.05 was used to determine whether the given combination of histone mark and cell line presented a significant change in proportion. Additionally, the base-2 logarithm of the odds ratio (OR) was used as a measure of effect size.

### Genomic region analysis

The probes in the microarray were assigned to a genomic region according to their position relative to the transcript information extracted from the R/Bioconductor package *TxDb.Hsapiens.UCSC. hg19.knownGene* (package version 3.0.0). A probe was said to be in a promoter region if it was located in a region up to 2 kb upstream of the transcription start site (TSS) of any given transcript. Similarly, a set of mutually exclusive regions were defined inside the transcripts, namely 5UTR, 3UTR, first exon, exon and intron. A probe could only belong to one of these categories, and when anyone overlapped with two or more of these regions in different transcripts, it was assigned to the region with the higher level of precedence (i.e. in the same order as stated above). If a probe was not assigned to any of these special regions, it was labeled by default as intergenic. A contingency table was built for each of the subsets, partitioning the complete set of probes according to membership of a given category and the subset of interest. A Pearson’s χ2 test was used to determine if there was a significant change in proportion between the number of probes marked as belonging to a given region inside and outside the subset of interest. A significance level of 0.05 and the effect size as measured by the odds ratio (OR) were employed for this test.

### CpG Island status analysis

The CpG island locations used in the analyses were obtained from the R/Bioconductor package *FDb.InfiniumMethylation.hg19* [66]. The generation procedure of these CpG Islands is described by Wu et al. [40] CpG shores were defined as the 2kbp regions flanking a CpG island. CpG shelves were defined as the 2kbp region either upstream or downstream of each CpG shore. Probes not belonging to any of the regions previously mentioned were assigned to the special category non-CpG island. Each probe was assigned to only one of the categories. A 4 × 2 contingency table was constructed for every subset of probes in order to study the association between the given subset and the different CpG island categories. A Chi squared test was used to determine whether any of the categories had a significant association with the given subset. For each of the CpG island status levels, a 2 × 2 contingency table was defined and another Chi squared test was used to independently evaluate the association of the given subset with each status level. A significance level of 0.05 was employed for all tests. Effect size was reported as the Odds Ratio for each of the individual tests.

### Gap distance analysis

Distance from both the centromere and telomere was measured for each of the probes in the Human Methylation450 microarray. In order to find significant differences between the probes inside the subset of interest and those in the background, a Wilcoxon non-parametric test was used. Again, a significance level of 0.05 was employed for all tests, and Cliff’s Delta (D) was used as a measure of effect size.

### Microarray background correction

Although it is sometimes referred to as a genome wide solution, the Infinium450 k microarray only covers a fraction of the entire genome. In its 27 k predecessor, the probes were mainly located at gene promoter regions, while in addition to the promoter probes, the Infinium450 k includes probes located inside genes and in intergenic regions [67]. The irregular distribution of probes can lead to unwanted biases when studying whether a selected subset of probes is enriched with respect to any functional or clinical mark. In this study, a reference to the background distribution of features was included in every type of statistical test performed in order to prevent the conclusions from being driven by the irregular distribution of probes. In qualitative tests (CpG island status, genomic region and histone mark enrichment), the contingency matrix was built to represent the background distribution of the microarray. Thus any significant result would indicate a departure from the fixed background distribution, and so avoid any manufacturer bias.

### Gene ontology analysis and annotation

Probe sets were converted to gene sets by using the annotation information present in the R/Bioconductor package *TxDb.Hsapiens.UCSC.hg19.knownGene* (*Carlson M. TxDb.Hsapiens.UCSC.hg19.knownGene: Annotation package for TxDb object(s).).* A probe was assigned to a gene if the probe was contained within the union of all the genomic regions represented by the different transcripts belonging to that gene, or in a 2kbp region upstream of the corresponding TSS. Probes converted in this way can be assigned to zero (intergenic probes) or more genes. After gene conversion, each subset of interest was analyzed using the HOMER software tool [68]. The software was configured to use the whole set of genes represented in the HumanMethylation450 architecture as a background. HOMER tested the genes in each subset of interest against 21 different databases, including the Gene Ontology (GO) Biological Process, Molecular Function and Cellular Component ontologies, as well as KEGG and Reactome pathway databases, among others.

## Conclusions

The present study provides a group of 6641 CpG sites that change their methylation levels from birth to 5 years of age in human blood leukocytes. Age-hypermethylated CpG sites are associated with genes related mainly to development, suggesting that DNA methylation-changes with age during the first years of life might play a significant role in the regulation of differentiation and leukocyte-specific functions. Conversely, genes with age-hypomethylated sites both reveal an immunological window of opportunity in childhood and indicate that blood leukocytes experience a genome remodeling, which is related to interaction with environmental factors. This underlines the importance of environmental exposures during the first years of life and highlights the need to take this into consideration in new strategies for disease prevention.

### Data access

The Illumina^®^ Infinium 450 k DNA methylation data sets from this study have been submitted to the NCBI Gene Expression Omnibus (GEO; http://www.ncbi.nlm.nih.gov/geo/) under accession number GSEXXXXX (SubSeries GSEXXXXX and GSEXXXXX).
